# Circadian clock regulates immune checkpoint inhibitor efficacy

**DOI:** 10.1016/j.apsb.2025.01.011

**Published:** 2025-01-23

**Authors:** Yining Niu, Motao Zhu

**Affiliations:** a CAS Key Laboratory for Biomedical Effects of Nanomaterials & Nanosafety, CAS Center for Excellence in Nanoscience, National Center for Nanoscience and Technology of China, Beijing 100190, China; b University of Chinese Academy of Sciences, Beijing 100049, China

**Keywords:** Clock disruption, Circadian rhythm, Anti-programmed death ligand 1, Myeloid-derived suppressor cells, Immunotherapy, Immune checkpoint inhibitors, *Bmal1*, Cancer treatment

Immunotherapy remains one of the most promising strategies for cancer treatment, with ICIs at the forefront of this innovation^1^. However, patient response to ICIs is highly variable, highlighting the urgent need to enhance their efficacy in clinical settings^2^. Addressing this challenge is critical for advancing the impact of immunotherapy in oncology.

Sleep, as a fundamental biological process, plays a crucial role in maintaining various physiological functions, including the regulation of circadian rhythms. The circadian clock plays a crucial role in regulating immune responses, influencing both host defence mechanisms and tumour immune surveillance^3^^,^^4^. A seminal study by Toda et al. identified *nemuri*, a sleep-related gene in *Drosophila*, establishing a critical link between sleep and immune function^5^. Subsequent research exploring the molecular mechanisms underlying circadian-immune interactions revealed a direct association between circadian clock disruptions and immune dysfunction in humans. Core circadian genes, such as *Clock*, *Bmal1*, *Per*, and *Cry*, are integral to the regulation of sleep patterns, duration, and quality. Dysregulation of these genes has been demonstrated to significantly impair immune responses^4^. The intricate relationship between the intestine, sleep, and the nervous system is multifaceted. Disruptions to systemic circadian rhythms, such as irregular light-dark cycles, have also been implicated in disturbances within the gut-brain axis, thereby compromising immune responses^6^. Studies have also indicated that the gut microbiota interacted with sleep through the “gut-brain axis”. Furthermore, the nervous system, particularly the ENS-often referred to as the “second brain”- is vital in regulating intestinal motility, secretion, and immune responses^7^. Overall, circadian rhythm disruptions, whether induced by *Bmal1* knockout or sleep deprivation, can negatively affect gut health by impairing the function of the ENS and altering the composition of gut microbiota. These changes, in turn, can affect sleep and nervous system function, compromising neuro-immune interactions, and increasing susceptibility to inflammation and immune-related disorders. While previous studies have established a causal relationship between the biological clock and immunity^8^^,^^9^, the precise mechanisms through which circadian rhythms modulate anti-tumour immune responses remain to be fully elucidated.

A recent study published in *Nature Immunology* has shed new light on this connection by uncovering a mechanism through which circadian rhythms regulate tumour immunosuppression *via* the modulation of MDSCs, thereby affecting the efficacy of ICIs^10^. Fortin et al.^10^ employed two distinct mouse models: a genetically engineered mouse model of CRC with either intact or disrupted biological clocks in intestinal epithelial cells, and a shift disruption mouse model with systemic clock disorders-to investigate how circadian rhythms interact with the tumour immune microenvironment.

By leveraging scRNA-seq analysis to profile the immune landscape in tumour tissues, the researchers observed an increased abundance of neutrophils and a decrease in cytotoxic CD8^+^ T cells in clock-disrupted mice compared to their WT counterparts. These findings were further validated by flow cytometry, showing consistent changes in the immune microenvironment following clock disruption. Notably, the total frequency of intestinal CD45^+^ immune cells remained unchanged, suggesting that clock disruption specifically impacts immune cell composition and function within the tumour microenvironment. To further characterize the role of the circadian clock in immune modulation, neutrophil- and monocyte-derived MDSCs were sorted using flow cytometry. Both clock disruption and tumours cause immune infiltration of MDSCs. These MDSCs exhibited elevated levels of reactive oxygen species, increased expression of the immune checkpoint molecule PD-L1, and upregulated immunosuppressive genes such as *S100a8*, *S100a9*, and *Wfdc17*, all of which suggest their heightened ability to suppress anti-tumour T cell responses (Fig. 1A). Notably, PD-L1-expressing MDSCs also contribute to immune regulation in various diseases beyond cancer, including Alzheimer's disease, Parkinson's disease, amyotrophic lateral sclerosis, and multiple sclerosis^11^, ^12^, ^13^. In Alzheimer's disease and Parkinson's disease condition, PD-L1-expressing MDSCs suppress T cell activation through the PD-1/PD-L1 pathway and induce the differentiation and the expansion of regulatory T cells with the presence of TGF-*β* and IL-10. However, in the later stages, the proliferation of MDSCs, especially the increase in monocytic MDSCs, may lead to the exacerbation of inflammation. These studies suggest that PD-L1-expressing MDSCs play a significant role in a variety of diseases and may represent potential targets for future therapeutic strategies.

**Figure 1:**
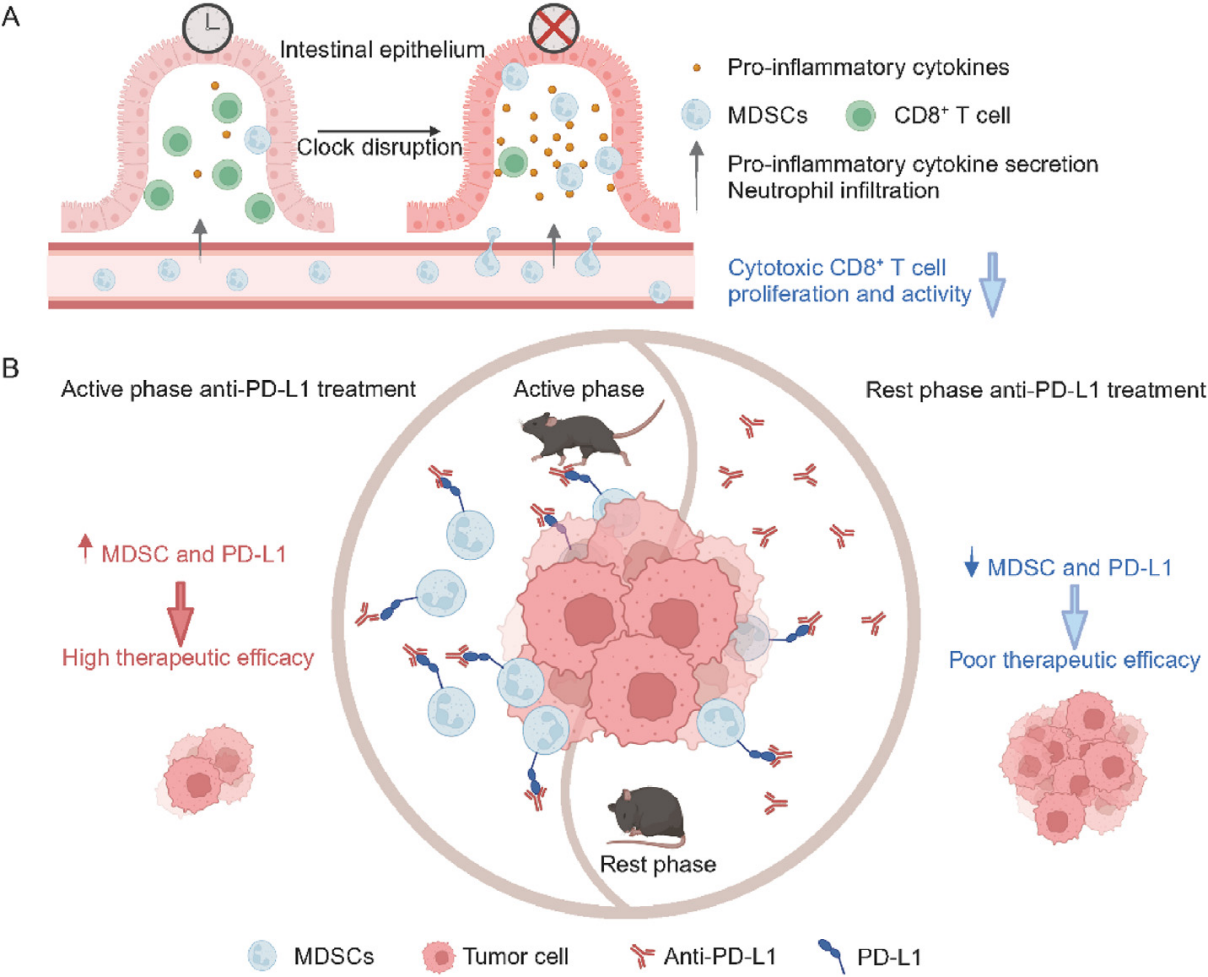
Circadian regulation of anti-PD-L1 blockade therapy through MDSCs. (A) Proposed model of normal *versus* clock-disrupted/Wnt-activated intestinal epithelium. (B) Model of circadian regulation of MDSCs for anti-PD-L1 treatment.

At the molecular level, Fortin et al.^10^ identified a signalling axis between the intestinal epithelial clock and immune cells. The researchers used WT and intestine-specific *Bmal1* knockout (*Bmal1*^*fl/fl*^*; Villin-Cre*) intestinal monolayers and organoids treated with Wnt3a and observed a Wnt-dependent transcriptional response. The Wnt target genes *c-Myc*, *Survivin*, and inflammatory cytokine *Cxcl5* were significantly upregulated in a Wnt3a-dependent manner in *Bmal1*^*fl/fl*^*; Villin-Cre* mice. This finding suggests that disruption of the circadian clock in intestinal epithelial cells promotes a pro-inflammatory response and hyperactivation of the Wnt signalling pathway. This hyperactivation drives the production of inflammatory mediators such as CXCL5, C-X-C motif chemokine ligand 6, and C-X-C motif chemokine ligand 2, which not only recruit neutrophils but also induce an immunosuppressive phenotype. The Wnt-dependent upregulation of MDSC-signature genes, including *S100a8*, *S100a9*, *Wfdc17*, and *arginase 2*, further supports this phenotype. Furthermore, transwell assays confirmed that neutrophil migration was significantly enhanced under conditions of clock disruption, especially in combination with Wnt pathway activation.

In addition to their findings in mouse models, Fortin et al.^10^ extended their research to human CRC samples using scRNA-seq data and observed a higher abundance of PD-L1-expressing myeloid cells in CRC tumours compared to normal colon tissue, further highlighting the clinical relevance of their discoveries. This finding raises a critical question: can circadian clock regulation affect the efficacy of anti-PD-L1 therapy in practice? To explore this, Fortin et al.^10^ used flow cytometry to assess the proportion of Gr1^+^ and PD-L1^+^ cells in the intestine and peripheral tissues of mice during both their early rest and active phases. Their findings revealed that the abundance of PD-L1-expressing MDSC follows a circadian rhythm, peaking during the early active phase. Strikingly, when anti-PD-L1 therapy was administered during this phase-when immunosuppressive MDSCs and PD-L1 expression were at their highest-the therapeutic efficacy improved significantly (Fig. 1B). This effect was observed not only in CRC models but also in subcutaneous models of CRC (MC38), lung cancer (CMT167), and melanoma (D4M-S).

Overall, this study emphasizes the intrinsic link between intestinal epithelial and immune cells. It underscores a previously unrecognized mechanism in which MDSCs are co-regulated by the circadian clock and Wnt signalling in the intestine. While previous studies have shown that clock disruption can enhance the recruitment of macrophages and regulatory T cells, this study expands the scope to include clock-dependent regulation of immunosuppression *via* MDSCs. By leveraging circadian control over immunosuppression, this work sets the stage for future clinical studies aimed at optimizing the timing for ICI delivery to maximize therapeutic benefits.

## Author contributions

Yining Niu: Writing-review & editing, Writing-original draft, Conceptualization. Motao Zhu: Writing-review & editing, Funding acquisition, Conceptualization. All of the authors have read and approved the final manuscript.

## Conflicts of interest

The authors have no conflicts of interest to declare.
